# The DNMT1-PAS1-PH20 axis drives breast cancer growth and metastasis

**DOI:** 10.1038/s41392-022-00896-1

**Published:** 2022-03-21

**Authors:** Yenan Fu, Xi Zhang, Xiao Liu, Peng Wang, Wenhui Chu, Wei Zhao, Yunling Wang, Guangbiao Zhou, Yu Yu, Hongquan Zhang

**Affiliations:** 1 Program for Cancer and Cell Biology, Department of Human Anatomy, Histology and Embryology, School of Basic Medical Sciences, PKU International Cancer Institute; MOE Key Laboratory of Carcinogenesis and Translational Research and State Key Laboratory of Natural and Biomimetic Drugs, Peking University Health Science Center, Beijing, 100191 China; 2 Department of Orthopedics, Peking University Third Hospital, Beijing, 100191 China; 3 Institute for Cardiovascular Research, Peking University, Beijing, 100191 China; 4 State Key Laboratory of Molecular Oncology, National Cancer Center, National Clinical Research Center for Cancer, Cancer Hospital Chinese Academy of Medical Sciences and Peking Union Medical College, Beijing, 100021 China

**Keywords:** Metastasis, Breast cancer

## Abstract

PH20 is a member of the human hyaluronidase family that degrades hyaluronan in the extracellular matrix and controls tumor progression. Inhibition of DNA methyltransferases (DNMTs) leads to elevated hyaluronan levels; however, whether DNMT inhibitors control PH20 remains unclear. Here, we report that the DNMT1 inhibitor, decitabine, suppresses PH20 expression by activating the long non-coding RNA PHACTR2-AS1 (PAS1). PAS1 forms a tripartite complex with the RNA-binding protein vigilin and histone methyltransferase SUV39H1. The interaction between PAS1 and vigilin maintains the stability of PAS1. Meanwhile, PAS1 recruits SUV39H1 to trigger the H3K9 methylation of PH20, resulting in its silencing. Functionally, PAS1 inhibits breast cancer growth and metastasis, at least partially, by suppressing PH20. Combination therapy of decitabine and PAS1-30nt-RNA, which directly binds to SUV39H1, effectively blocked breast cancer growth and metastasis in mice. Taken together, DNMT1, PAS1, and PH20 comprise a regulatory axis to control breast cancer growth and metastasis. These findings reveal that the DNMT1-PAS1-PH20 axis is a potential therapeutic target for breast cancer.

## Introduction

RNA-binding proteins are important regulators of mRNAs and non-coding RNAs in cancer. Vigilin, also called high-density lipoprotein binding protein Hdlbp, is a highly conserved RNA-binding protein, containing 15 tandem KH domains. It could bind over 700 RNAs to regulate mRNA translation, transport, and stability.^1^ In addition, vigilin also interacts with histone methyltransferase SUV39H1 to participate in heterochromatin formation.^2^ However, whether vigilin targets long non-coding RNAs (lncRNAs) and influences their stability are unknown. LncRNAs are transcripts with more than 200 nucleotides, lacking protein-coding potential.^3,4^ Although thousands of lncRNAs have been identified, only a small number of them are well characterized in cancer progression.^5,6^ The lncRNA PHACTR2-AS1 (also known as NR027113 and LncIHS) was originally found to promote cell proliferation and invasion in hepatocellular carcinoma.^7,8^ However, PHACTR2-AS1(PAS1) plays the opposite role in breast cancer. PAS1 expression is suppressed in breast cancer and lower PAS1 level predicts poor outcomes for breast cancer patients.^9^ PAS1 directly binds to ribosome DNA and inhibits ribosome synthesis, ultimately blocking breast cancer development.^9^ Nevertheless, the precise mechanism by which PAS1 attenuates breast cancer metastasis is unclear.

Hyaluronan is critical for forming the extracellular matrix and mediating cell to cell interactions.^10,11^ Hyaluronidases are responsible for degrading hyaluronan; thus, they play important roles in cancer progression.^12^ The family of hyaluronidases includes Hyal-1, Hyal-2, Hyal-3, Hyal-4, PH20, and a pseudogene in the human genome.^13^ Hyal-1, Hyal-2, and PH20 have hyaluronidase activity for hyaluronan hydrolysis.^14^ The hyaluronidase family members have different expression profiles in different cancers. Hyal-1 is significantly overexpressed in bladder cancer and has been used as a diagnostic marker for bladder cancer.^15^ The activities of Hyal-1, 2, 3, and PH20 are increased in colorectal cancer, and higher levels of Hyal-1 and Hyal-2 expression correlate with more aggressive stages.^16^ In breast cancer, Hyal-1 acts as a tumor promoter, and its overexpression promotes cancer cell growth and motility.^17^ Moreover, Hyal-2 is also implicated in the invasiveness of breast cancer cells.^18^ Hyal-1 and Hyal-3 have elevated expression in lung cancer cells.^19^ PH20 (also called SPAM1) is specifically expressed on the surfaces of sperm and participates in the process of sperm-egg fusion.^20^ Aberrant expression of PH20 is involved in the progression of prostate, laryngeal, and breast cancers.^21–23^ However, the mechanism through which PH20 expression is regulated in cancer remains unknown.

DNA methyltransferases (DNMTs) are key enzymes that maintain DNA methylation.^24^ DNMT inhibitors can induce the re-expression of silenced tumor suppressors by decreasing DNA methylation levels.^25^ There are two FDA-approved DNMT inhibitors, 5-aza-2′-deoxycytidine (decitabine) and 5-aza-cytidine (azacitidine), which act as nucleoside analogs for incorporation into newly synthesized DNA strands.^26^ These two agents have been successfully used to treat myelodysplastic syndrome (MDS) and myeloid hematologic malignancies. However, the efficacy of DNMT inhibitors in the treatment of solid tumors remains unclear.^27^ In this study, we reported that a DNMT1 inhibitor controls PH20 expression by activating PAS1. The combination therapy of DNMT inhibitor decitabine and PAS1-30nt-RNA efficiently blocked breast tumor growth and metastasis.

## Results

### DNMT1 suppresses PAS1 expression in breast cancer cells

**Fig. 1:**
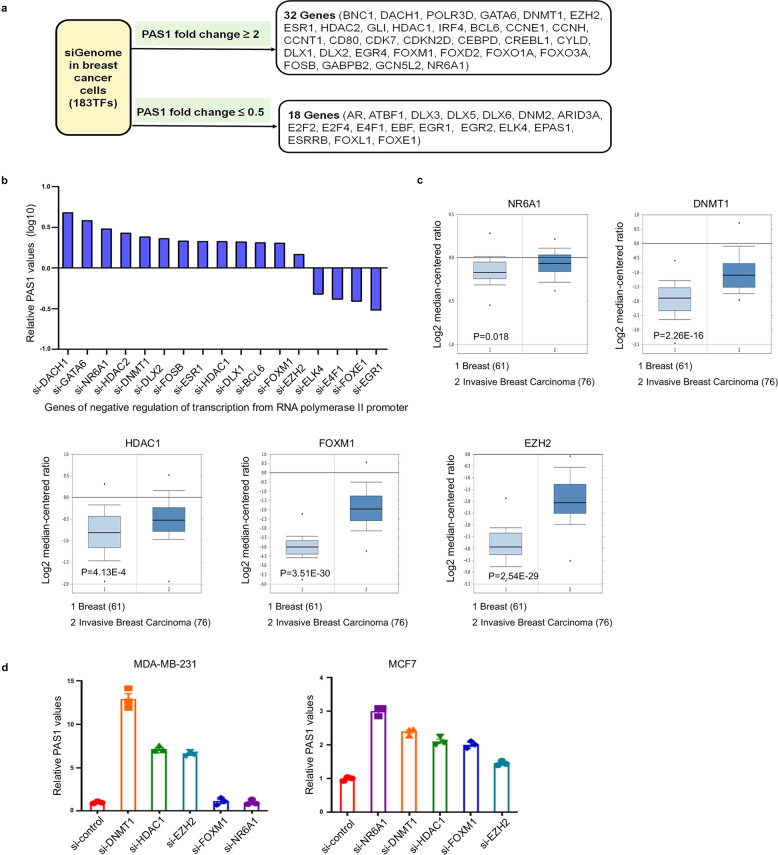
Screening the transcript factors (TFs) that regulate PAS1. **a** Flow chart depicts the screening process. siRNAs of 183 TFs were transfected into breast cancer cell MCF7. After 72 h, total RNA was extracted, followed by qRT-PCR to detect the level of PAS1. **b** The TFs were filtered out from the term of negative regulation of transcription from RNA polymerase II promoter, and the changes of PAS1 levels were shown. **c** Analysis of expression of five TFs in breast cancer patients’ samples obtained from Oncomine datasets. **d** Control and siRNAs were transfected into MDA-MB-231 and MCF7 cells. After 48 h, total RNA was extracted, followed qRT-PCR analysis

To scan the transcriptional regulation of PAS1, the small interfering RNAs (siRNAs) targeting 183 transcription factors (TFs) were transfected into MCF7 cells, and the fold changes of PAS1 were determined by RT-PCR. We found that 32 TF siRNAs resulted in the activation of PAS1 (fold change ≥2), whereas 18 TF siRNAs led to the suppression of PAS1 (fold change ≤0.5) (Fig. 1a). GO analysis was then performed using the 50 genes with significant changes in PAS1 level (Supplementary Fig. S1a). Given the suppressive role of PAS1 in breast cancer, we focused on the 17 genes involved in the negative regulation of transcription from RNA polymerase II promoter. Among these genes, 13 siRNAs were found to upregulate PAS1, indicating that these 13 genes could suppress PAS1 transcription (Fig. 1b). Furthermore, analysis of oncomine datasets showed that mRNA expression of 5 genes from the 13 genes was higher in breast cancer tissues than that in normal breast tissues, including NR6A1, DNMT1, HDAC1, FOXM1, and EZH2 (Fig. 1c and Supplementary Fig. S1b). We verified their mRNA level in breast cancer cell lines by RT-qPCR, and found that DNMT1 knockdown led to an obvious upregulation of PAS1 (Fig. 1d). Thus, we prioritized DNMT1 as a key gene in regulating PAS1.

**Fig. 2:**
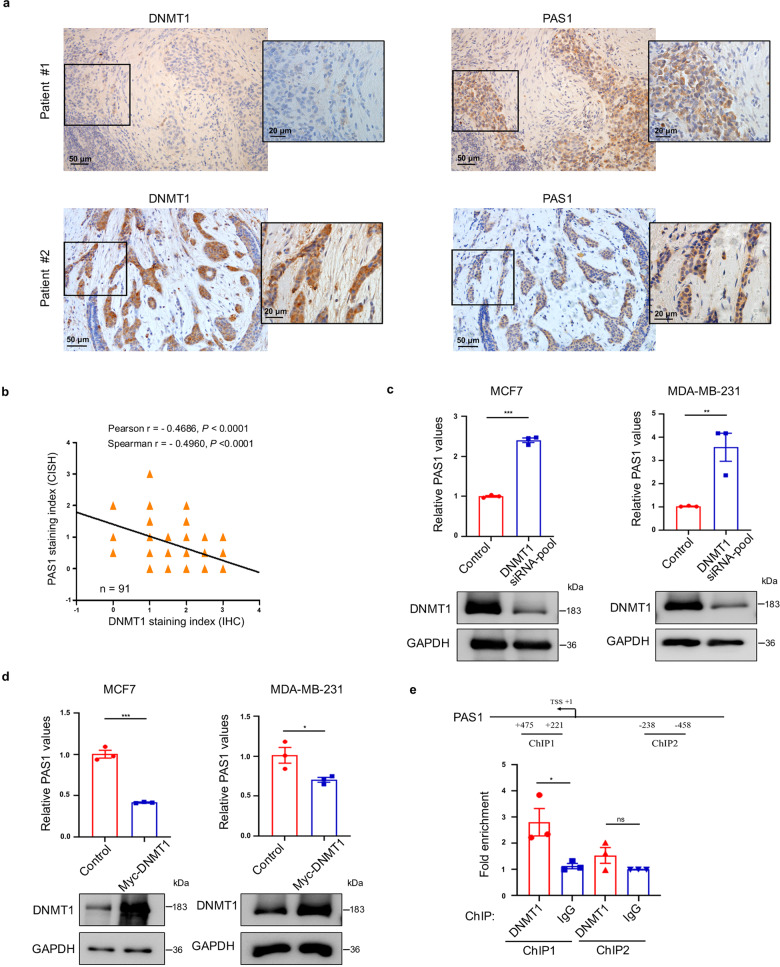
DNMT1 suppresses PAS1 expression. **a**, **b** Both PAS1 expression (chromogenic in situ hybridization, CISH) and DNMT1 expression (immunohistochemistry, IHC) were examined in paraffin-embedded tissues of 91 breast cancer patients. Representative images are shown in (**a**). Pearson and Spearman correlation tests were performed in (**b**). **c** Control and DNMT1 siRNAs pool was transfected, followed qRT-PCR and Western blot analysis. **d** Empty vector or Myc-DNMT1 was transfected, followed qRT-PCR and Western blot analysis. **e** Schematic representations of the region used for PAS1 ChIP assay. Lysates of Hs578T cells were extracted for ChIP assays using anti-DNMT1 antibodies. Q-PCR assays were performed to quantify ChIP-enriched DNAs. Data in **c**–**e** are mean ± SEM from three experiments by two-tailed *t* test. *p < 0.05, **p < 0.01, ***p < 0.001

To explore the relationship between DNMT1 and PAS1 expression, we firstly examined the PAS1 RNA level by RNA in situ hybridization and DNMT1 protein levels by immunohistochemical staining of human breast cancer tissue arrays. As expected, the expression of DNMT1 was negatively correlated with that of PAS1 (Fig. 2a, b). The level of PAS1 was higher in the group with low DNMT1 expression than in the group with high DNMT1 expression. Likewise, the level of DNMT1 was higher in the group with low expression of PAS1 than in the group with high expression of PAS1 (Supplementary Fig. S2a), indicating that DNMT1 levels are negatively correlated with PAS1 levels in breast cancer tissues.

To verify the regulation of DNMT1 on PAS1, we examined the levels of PAS1 upon knockdown or overexpression of DNMT1. DNMT1 knockdown resulted in an upregulation in the expression of PAS1 (Fig. 2c and Supplementary Fig. S2b), whereas overexpression of DNMT1 led to a reduction in PAS1 RNA levels (Fig. 2d). To further understand the regulation of DNMT1 on PAS1, we observed the occupancy of DNMT1 at the PAS1 promoter and found that DNMT1 could bind to the PAS1 promoter (Fig. 2e and Supplementary Fig. S2c). Taken together, these data indicate that the transcription of PAS1 is negatively regulated by DNMT1.

### Vigilin enhances PAS1 stability by interacting with PAS1

**Fig. 3:**
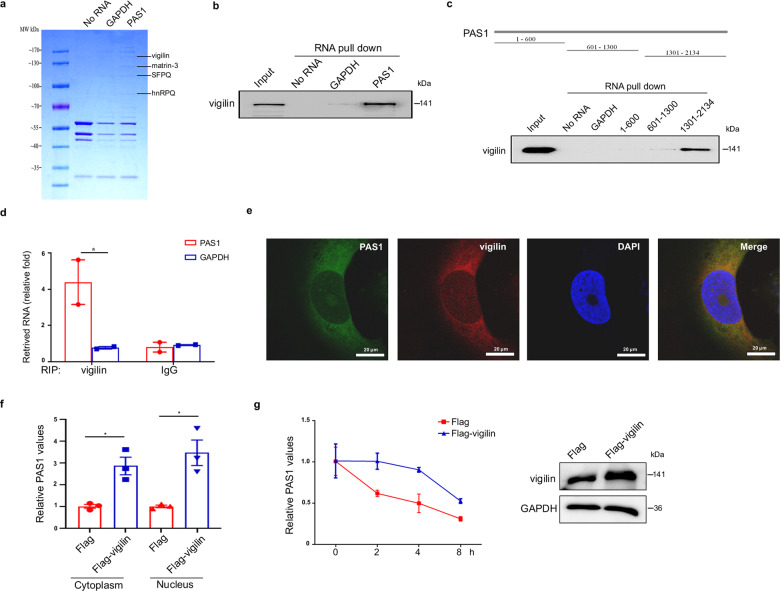
Vigilin interacts with PAS1 and maintains its stability. **a** RNA pull down combined with mass spectrometry was performed using biotin-labeled PAS1 RNA. Biotin-labeled GAPDH mRNA were used as negative controls. **b** Biotin-labeled PAS1 and GAPDH were incubated with lysates from MCF7 cells, followed by RNA pull down. **c** Biotin-labeled RNAs containing different regions of PAS1 were designed. RNA pull down was performed by incubating biotin-PAS1 fragments with MCF7 cell lysates. **d** RIP assay was performed with anti-vigilin antibodies. RIP enrichment was determined as the amount of RNA recovered in association with vigilin, relative to that recovered with an IgG control. Data are mean ± SEM by two-tailed *t* test. *p < 0.05. **e** Co-staining of PAS1 (RNA FISH, Alexa Flour 488), vigilin (immunofluorescence, Alexa Flour 568) and nuclei (DAPI) in Hs578T cells. **f** Flag or Flag-vigilin was transfected into Hs578T cells and both cytoplasmic and nuclear RNA was then extracted for qRT-PCR. Data are mean ± SEM by *t* test. **g** Flag or Flag-vigilin was transfected into Hs578T cells. After 48 h, cells were treated with 20 μg/ml ActD. Total RNA was extracted at different time point for qRT-PCR. Data are mean ± SD by *t* test. Pro*t*ein was extracted at 0 h for Western blot

To determine the protein interactome of PAS1, RNA pull-down combined with mass spectrometry was performed. Four candidates are found including vigilin, matrin-3, SFPQ, and hnRPQ (Fig. 3a and Supplementary Table. S1). To verify these candidates, we carried out RNA pull-down combined with western blot analysis. Results showed that the most obvious and specific PAS1-interacing protein is vigilin (Supplementary Fig. S3 and Fig. 3b). We then constructed three PAS1-truncation mutants and found that the C terminus from nucleotides 1301 to 2134 was sufficient for vigilin binding (Fig. 3c). The interaction between PAS1 and vigilin was then examined in vivo using RNA immunoprecipitation (RIP). As expected, antibodies against vigilin precipitated endogenous PAS1 in MCF7 cells (Fig. 3d). Furthermore, we observed co-localization of PAS1 and vigilin in both the nucleus and cytoplasm of Hs578T cells by RNA FISH and immunofluorescence staining (Fig. 3e). These data indicated that the endogenous PAS1-vigilin complex did indeed exist. Given the function of vigilin in maintaining mRNA stability, we speculated whether vigilin affected the stability of PAS1. To substantiate this, we first detected the effect of vigilin on PAS1 levels, and found that overexpression of vigilin led to an increase of PAS1 in both the nucleus and cytoplasm (Fig. 3f). Next, Hs578T cells were treated with the transcriptional inhibitor actinomycin D (ActD) to detect the half-life of PAS1. The results showed that the half-life of PAS1 was approximately 4 h in control cells. After vigilin overexpression, the half-life of PAS1 was prolonged to 8 h (Fig. 3g). These data suggest that PAS1 interacts with the RNA-binding protein vigilin to maintain its stability.

### PAS1 downregulates PH20 expression by H3K9 trimethylation

**Fig. 4:**
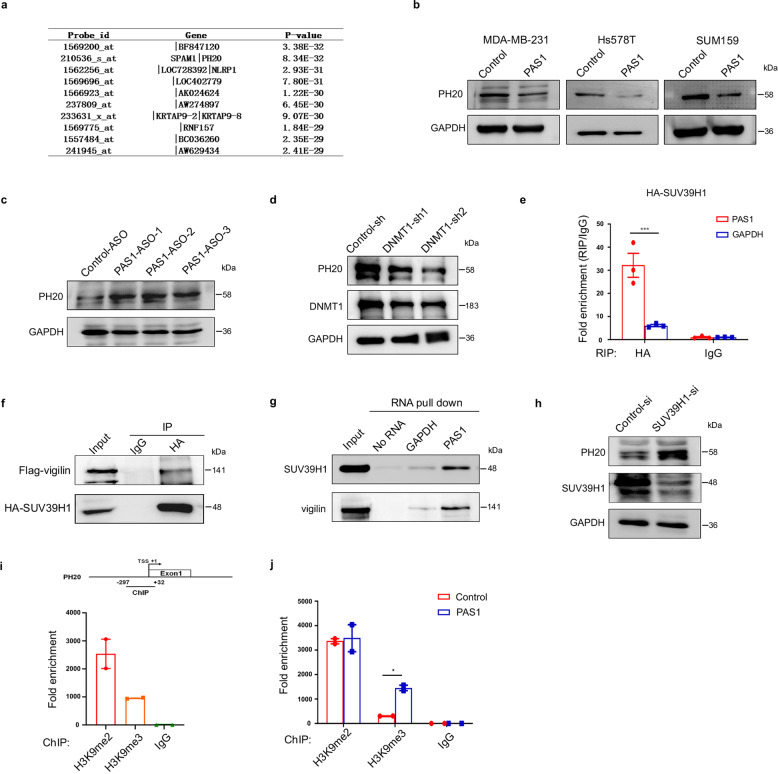
PAS1 suppresses PH20. **a** The top 10 genes from co-expression analysis were showed. **b** An empty or PAS1-overexpressing vector was transfected into different breast cancer cells, followed by Western blot. **c** Control or PAS1 ASOs were transfected into MCF7 cells, followed by Western blot. **d** Lysates were prepared from MDA-MB-231-control/DNMT1-shRNAs cells for Western blot analysis. **e** HA-SUV39H1 was transfected into HEK293A cells. RIP assay was performed using anti-HA antibodies, followed by qPCR to detect PAS1 and GAPDH (negative control). **f** Vectors expressing HA-SUV39H1 and Flag-vigilin were transfected into HEK293A cells, followed by Co-IP assay using anti-HA antibodies. **g** Biotin-labeled PAS1 was incubated with lysates from MCF7 cells for RNA pull down analysis. **h** SUV39H1 siRNA was transfected into MDA-MB-231 cells, followed by Western blot. **i** SUV39H1 was transfected into HEK293A cells. Lysates were extracted for ChIP assays using anti-H3K9me2 and anti-H3K9me3, followed by qPCR to quantify ChIP-enriched DNAs using specific primer for PH20 promoter region. **j** Lysates were extracted from stable Hs578T-control/PAS1 cells for ChIP assays

To explore the target genes of PAS1, we performed a co-expression analysis of PAS1. We downloaded 100 gene expression profiles containing PAS1 Affy-probe from GEO datasets. By calculating the correlation between PAS1 probe and other probes, the correlation matrix was obtained, and finally, the *P*-value was calculated using the Rank method (Supplementary Table. S2). The top 10 genes with the smallest *P* value were shown in Fig. 4a. Among the 10 genes, the top 2 gene SPAM1 (Sperm adhesion molecule 1, also known as PH-20) has hydrolase activity. Aberrant expression of PH20 has been found in multiple cancers and has been implicated in cancer metastasis.^21,23^ Thus, PH20 was chosen as the candidate PAS1 target. We first estimated the levels of PH20 with or without PAS1 overexpression. The results showed that PAS1 overexpression inhibited PH20 expression (Fig. 4b and Supplementary Fig. S4a). In contrast, PAS1 knockdown restored the expression of PH20, indicating the negative regulation of PAS1 on PH20 (Fig. 4c and Supplementary Fig. S4b). Given the suppression of DNMT1 on PAS1, we further estimated the effect of DNMT1 on PH20 levels. DNMT1 knockdown resulted in a decrease in PH20 expression (Fig. 4d and Supplementary Fig. S4c), suggesting that the DNMT1-PAS1-PH20 cascade occurred in breast cancer cells.

Given that PAS1 directly binds to histone methyltransferase SUV39H1,^9^ which also interacts with vigilin,^2^ the three molecules may form a complex. To test this idea, RIP and Co-IP assays were performed and identified that both PAS1 and vigilin could be immunoprecipitated by SUV39H1 (Fig. 4e, f). Next, RNA pull-down showed that biotin-labeled PAS1 RNA could bind to both SUV39H1 and vigilin (Fig. 4g), suggesting that the three molecules form a tripartite complex. Since SUV39H1-catalyzed H3K9 dimethylation and trimethylation (H3K9me2 and H3K9me3) are known to mediate gene silencing,^28^ SUV39H1 may participate in the suppression of PAS1 on PH20. Indeed, SUV39H1 knockdown enhanced the expression of PH20 (Fig. 4h and Supplementary Fig. S4d). Both H3K9me2 and H3K9me3 were enriched in the PH20 promoter (Fig. 4i). Overexpression of PAS1 markedly promoted the occupancy of H3K9me3 (Fig. 4j), suggesting that H3K9me3 is involved in the suppression of PAS1 on PH20. These data suggest that PAS1 promotes the occupancy of H3K9me3 at the PH20 promoter by interacting with SUV39H1, leading to the suppression of PH20.

### PAS1 inhibits breast cancer cells growth and metastasis partially through PH20

**Fig. 5:**
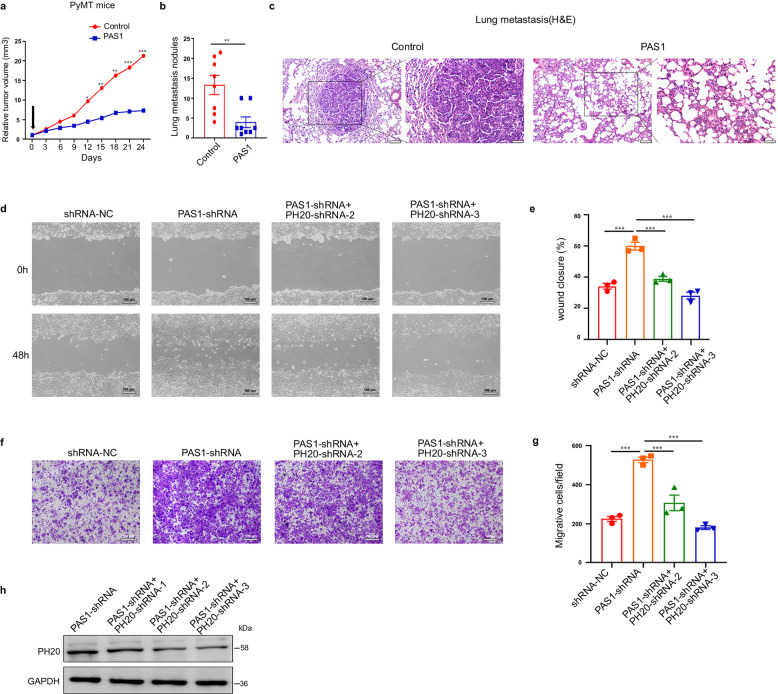
PAS1 inhibits cancer cell growth and metastasis. **a** Control or PAS1-expressing lentiviruses were orthotopically injected into multiple mammary glands of PyMT mice. Tumor-growth curves were derived by measuring tumor volume once every 3 days Data are mean of tumor volume (control group, *n* = 45 tumors; PAS1 group, *n* = 46 tumors). **b** Lung-metastatic nodules were counted (*n* = 8/group). Data are means ± SEM by two-tailed *t* test. **c** Representative lung-metastasis specimens were sectioned and stained with H&E. **d**–**g** PH20 shRNAs were transfected into stable MCF-7-control/PAS1-shRNA cells. Wound healing scratch and migration assays were performed in the stable cells. Data are from three experiments by two-tailed *t* test. **h** The efficacy of PH20 shRNAs was detected by Western blot

Lentiviruses carrying a control sequence or PAS1 were orthotopically injected into multiple mammary glands of 12-week-old female PyMT-transgenic mice, a widely used breast cancer model. The efficiency of infection is about 50% by observing GFP fluorescence using freezing section (Supplementary Fig. S5a). The results showed that lentiviruses carrying PAS1 reduced tumor volumes in PyMT mice bearing multiple tumors, and decreased the number of lung metastatic nodules (Fig. 5a, b). Lung metastases were verified by histological staining (Fig. 5c). To further observe the role of PAS1 in regulating metastasis of breast cancer cells, control cells and PAS1-overexpressing cells were orthotopically implanted into the abdominal mammary fat pad of NOD/SCID mice. After 8 weeks, the mice were subjected to bioluminescence imaging using the IVIS imaging system. The results showed that PAS1 overexpression led to decreased tumor volumes and weakened liver metastasis of MDA-MB-231-Luc-D3H2LN cells (Supplementary Fig. S5b–d). These data demonstrated that PAS1 overexpression inhibited the growth and metastasis of breast cancer cells in vivo. Next, the role of PAS1 in suppressing cancer cell migration was confirmed in vitro by wound healing and cell migration assays. PAS1 knockdown promoted breast cancer cells migration. However, after PH20 was concurrently knocked down, the velocity of wound closure and cell migration were restored to the levels of that of the control cells (Fig. 5d–h), suggesting that PH20 is involved in the suppression of PAS1 on cancer cell migration.

### Combination of PAS1-30nt-RNA and DNMT inhibitor suppresses breast cancer

**Fig. 6:**
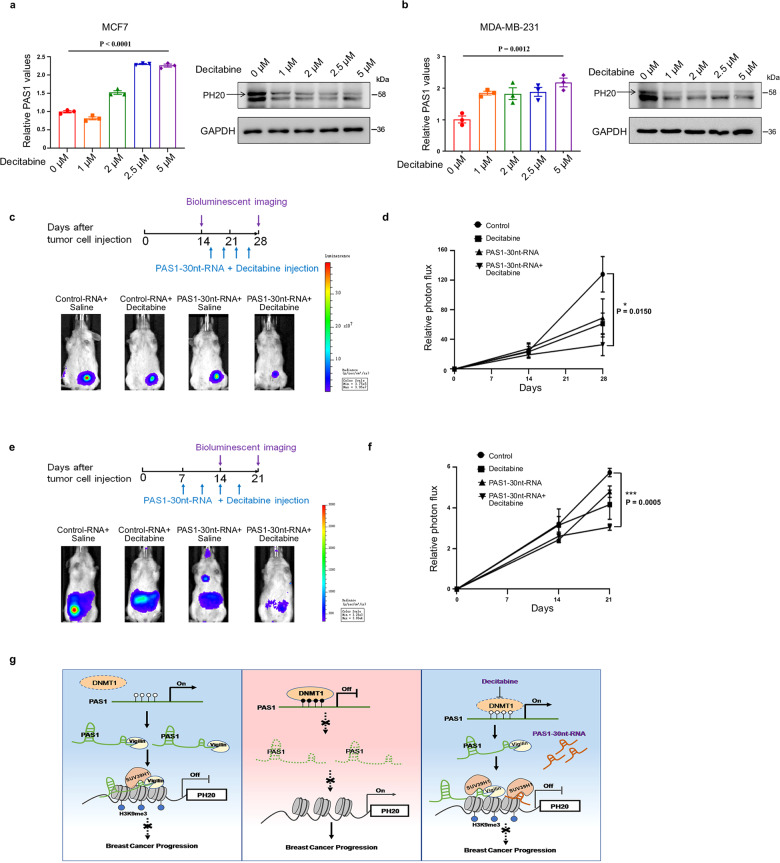
Combination treatment of PAS1-30nt-RNA and decitabine inhibits tumor growth and metastasis. **a**, **b** Cells were treated with different doses of decitabine for 48 h, followed by RT-qPCR and Western blot. Data represent the means ± SEM by one-way ANOVA analysis. **c** MDA-MB-231-Luc-D3H2LN cells (2 × 10^6^ cells) were inoculated onto the mammary fat pad of mice. After 2 weeks, the mice were divided into four groups (*n* = 4/group), and followed the injection of PAS1-30nt-RNA or/and decitabine. Representative in vivo bioluminescence images. **d** Bioluminescence-based quantitation of primary tumor sizes. Data are means ± SEM by two-tailed *t* test. **e** MDA-MB-231-Luc-D3H2LN cells (1.5 × 10^6^ cells) were injected into the tail vein of mice. After 1 weeks, the mice were divided into four groups (*n* = 3/group), and followed the injection of PAS1-30nt-RNA or/and decitabine. Representative in vivo bioluminescence images. **f** Bioluminescence-based quantitation of metastatic signals. Data are means ± SEM by two-tailed *t* test. **g** A working model

DNMTs are promising therapeutic targets, and some DNMT inhibitors, such as decitabine, have been used to treat cancer. In light of the above-mentioned effect of DNMT1 knockdown on PAS1 and PH20, we evaluated the action of decitabine in breast cancer cells. Consistently, administration of decitabine resulted in elevated PAS1 levels and decreased PH20 levels (Fig. 6a, b). Some studies have confirmed that combinations of DNMT inhibitors with other drugs appear to be more effective than single drug. PAS1-30nt-RNA is a synthesized PAS1 fragment with modifications of 2′-O-methylation and 5′-Cholesterol for in vivo RNA delivery. Accordingly, we used mice that harbored xenografts to observe whether decitabine alone, PAS1-30nt-RNA alone, or in combination with PAS1-30nt-RNA can suppress tumor growth in vivo. The combination of PAS1-30nt-RNA and decitabine was more efficient than decitabine alone or PAS1-30nt-RNA alone in inhibiting tumor growth (Fig. 6c, d). The body weights of mice in the four groups has not obvious changes (Supplementary Fig. S6a). We also analyzed the cellular function affected by the treatment on tumor tissue sections. The combination treatment resulted in a decrease in Ki67 expression and an increase in Bax expression compared to other three groups (Supplementary Fig. S6b). It indicated that the treatment of PAS1-30nt-RNA and decitabine inhibited tumor cell proliferation and promoted cell apoptosis.

Next, we observed the therapeutic effect of decitabine and PAS1-30nt-RNA on the metastatic mice models. As expected, the combination group showed a less metastatic signals than decitabine alone or PAS1-30nt-RNA alone group (Fig. 6e, f). In addition, since the PAS1-PH20 can regulate migration, the PAS1-30nt-RNA and Decitabine combination treatment was also used on cells in vitro. Migration assays were performed in the 4 group cells, and indicated that the combination treatment significantly inhibited cancer cell migration (Supplementary Fig. S6c). These results suggest that the combination of decitabine and PAS1-30nt-RNA is of therapeutic value for inhibiting breast cancer growth and metastasis.

## Discussion

Here, DNMT1 was identified as an upstream regulator of PAS1. The depletion of DNMT1 suppresses the expression of hyaluranidase PH20 by activating PAS1. PAS1 interacts with vigilin to maintain its stability. PAS1 also recruits SUV39H1 to the PH20 promoter, leading to the enrichment of H3K9 tri-methylation, which turns off the expression of PH20. However, after PAS1 was suppressed by DNMT1 overexpression, PH20 expression was activated, which subsequently promoted breast cancer progression. Administration of decitabine and PAS1-30nt-RNA restored the suppression of PH20, blocking cancer progression (Fig. 6g).

Vigilin is found to interact with lncRNA PAS1 to enhance its stability. Likewise, vigilin also increases the stability of vitellogenin mRNA by binding to the its 3′ UTR.^29^ Unlike PAS1 and vitellogenin, vigilin reduces the mRNA stability of CSF-1R.^30^ The different role that vigilin plays may be due to the competition of vigilin with another RNA-binding proteins to regulate the stability of target RNAs. Since RNA-binding protein HuR could stabilize CSF-1R mRNA,^31^ vigilin competes with HuR to bind to CSF-1R mRNA leading to the decrease of CSF-1R stability. PAS1 indirectly suppresses CSF-1R mRNA by interacting with vigilin (Supplementary Fig. S7), suggesting that CSF-1R mRNA may also be a target of the PAS1-vigilin complex in breast cancer.

The three molecules of PAS1, SUV39H1, and vigilin could form a complex. However, vigilin could not facilitate the binding of SUV39H1 and PAS1 (Supplementary Fig. S8a), and similarly, PAS1 had no effect on the binding of vigilin and SUV39H1 (Supplementary Fig. S8b). The role of vigilin in the complex is to stabilize PAS1, and SUV39H1 is responsible for the suppression of PAS1 on PH20. Since DNMT1 is an upstream regulator of PAS1, treatment with decitabine led to a decrease in PH20 levels, which suggests that DNMT inhibitors can increase hyaluronan levels in the extracellular matrix. Consistently, Kohiand colleagues reported that hyaluronan levels in the culture supernatant of PDAC cells are significantly increased in response to treatment with a DNMT1 inhibitor.^32^ Thus, PAS1 knockdown may promote the degradation of hyaluronan by upregulating PH20 to facilitate cancer cell migration, which provides a novel mechanism through which PAS1 blocks cancer metastasis. The direct regulation of the DNMT1-PAS1-PH20 cascade on hyaluronan levels will be explored in further studies.

The enrichment of DNMT1 on PAS1 is modest even if detected by a highly sensitive qPCR. Other factors may be involved in the recruitment of DNMT1 to the PAS1 promoter. EZH2 and YY1 could bind to PAS1 promoter to suppressing its expression.^9^ In fact, DNMT1 can be recruited by EZH2 to form a complex to silence gene expression.^33^ Thus, other factors may mediate the recruitment of DNMT1 to the PAS1 promoter, such as EZH2 and YY1.

Given the action of DNA methylation in cancer and the reversibility of epigenetic modifications, DNMT inhibitors have a strong potential for use in cancer therapy. Combination therapy with a DNMT inhibitor and a histone deacetylase inhibitor has shown encouraging results.^34,35^ In addition, the synergistic effects of DNMT inhibitors and chemotherapeutic agents have also been observed.^36^ However, the high toxicity of DNMT inhibitors for long-term use is a main factor limiting their clinical application.^37^ In the past 10 years, RNA molecule has become one of the most promising therapeutic targets of cancers. Currently, lncRNA-based therapeutics are focused on applying specific antisense oligonucleotides (ASOs) against lncRNAs. We apply another strategy to restore RNA level with synthetic RNA molecules. We synthesize a functional fragment (PAS1-30nt-RNA) of a lncRNA (PAS1) and add chemically modifications for in vivo RNA delivery. PAS1-30nt-RNA naturally exists in epithelial cells and thus has rather low toxicity. The combination of decitabine with PAS1-30nt-RNA showed a significant anti-tumor activity superior to that of decitabine alone. The marked efficacy of the combination not only depends on the synergistic effects of the DNMT inhibitor and PAS1-30nt-RNA, but also on the enhanced expression of PAS1 induced by the DNMT inhibitor. Thus, an investigation of the combination of DNMT inhibitors with small-molecule RNA might be expected in the future.

## Materials and methods

### Cell culture

Human breast cancer cells were obtained from the Cell Resource Center, Peking Union Medical College (the Headquarters of National Infrastructure of Cell Line Resource, NSTI). The species origin of the cell lines was confirmed with PCR. The identity of the cell lines was authenticated with short tandem repeat profiling. The cell lines were checked free of mycoplasma contamination by PCR. Cells were grown in DMEM medium (Gibco) supplemented with 10–15% fetal bovine serum at 37 °C under 5% CO_2_ in a humidified incubator.

### RNA isolation and quantitative real-time PCR (qRT-PCR)

Total RNA was extracted using TRIzol reagent (Invitrogen). RNA nuclear or cytoplasmic fraction was performed using the Cytoplasmic & Nuclear RNA Purification Kit (#21000, Norgen, BiotekCorporation, CA) according to the manufacturer’s instructions. A HiScript II Q RT SuperMix Kit (Vazyme, China) was used to synthesize the cDNA. A ChamQ SYBR qPCR Master Mix (Vazyme, China) was used for real-time PCR with a Light Cycler 96 detection system (Roche). Primer sequences:

PAS1-F-GTCAGGCTCCTGGATCTTCTAG; R-AAGGGCTAAGGAACTCGATTG.

### RNA pull-down assay

Plasmid of pcDNA3.1-PAS1 was linearized as DNA templates. Biotin-labeled RNA was produced with the MEGAscript T7 Kit (Ambion) with biotin-16-UTP (Ambion). The biotin-labeled RNA was further purified using the MEGA clear Kit (Ambion). 10 μg biotin-labeled RNA in RNA structure buffer (10 mM Tris pH7, 0.1 M KCl, 10 mM MgCl_2_) was heated to 95 °C for 2 min, on ice for 3 min, and then at RT for 30 min. Then RNA was incubated with pre-cleared cell lysates (containing 1 mg proteins) in 500 μl binding buffer (150 mM KCl, 25 mM Tris pH 7.4, 0.5% DTT, 0.5% NP40, 1 mM EDTA, 2 U/ml RNasin) and then incubated at RT for 1 h. Next, 25 μl of washed streptavidin Dynabeads (Invitrogen) was added to each binding reaction and incubated at RT for 1 h. The streptavidin Dynabeads were washed with binding buffer for 5 times and boiled in SDS buffer. The retrieved proteins were analyzed by Western blot.

### RNA immunoprecipitation (RIP) assay

EZ-Magna RIP RNA-Binding Protein Immunoprecipitation Kit (Millipore) was used to carry out the RNA immunoprecipitation assay. According to the manufacturer’s instructions 5 μg of anti-FLAG, anti-Vigilin, anti-mouse IgG, or anti-rabbit IgG antibody was used. The co-precipitated RNAs were extracted, and RT-PCR was performed to analyze the samples.

### Chromatin immunoprecipitation

A chromatin immunoprecipitation (ChIP) assay was performed using the SimpleChIP^®^ Enzymatic Chromatin IP Kit (#9003, Cell Signaling Technology). The cells were cross-linked for 10 min at 37 °C with 1% formaldehyde added directly into the culture medium. Cross-linking was quenched by the addition of glycine to a final concentration of 0.125 M, followed by incubation at RT for 5 min. Cells were then lysed, and the cytoplasmic fraction was discarded. Nuclear fractions were sonicated to obtain 300–500 bp DNA fragments. After centrifugation, the supernatant was transferred to a new tube. The pre-cleared supernatant was immunoprecipitated with anti-H3K9me2 (Cell Signaling Technology), anti-H3K9me3 (Cell Signaling Technology), and anti-DNMT1 (Abcam) antibodies overnight at 4 °C, with rotation, followed by incubation with protein G-conjugated magnetic beads for 2 h at 4 °C. After elution and reversal of cross-linking, the precipitated DNA was purified and resuspended in Tris-EDTA for qPCR. The primer sequences used for qPCR are listed below:

PAS1 CHIP1 F-CAGTAAGTAAGGCAGTGAGC; R-CTGCGTCAGATGAAGAAGA;

PAS1 CHIP2 F-TGGCATCTGCTTGGC; R-GAGGTGTTTGCGTCAT;

PH20 CHIP F-AAGAGGCAGCATTTGA; R- AGGTGGAGCCAGTCAT.

### RNA in situ hybridization

Cells were briefly rinsed with phosphate-buffered saline (PBS) and fixed with 4% formaldehyde for 15 min at room temperature, followed by permeabilization with pepsin (0.1% in 10 mM HCl) for 1 min at 37 °C. After washing, the cells were dehydrated using 70, 80, 90, and 100% ethanol, then air dried. A 5′biotin-labeled LNA^TM^-modified PAS1 probe (Exiqon, Vedbaek, Denmark) was diluted in hybridization buffer (50% deionized formamide, 50 mM sodium phosphate pH 7, 10% dextran sulfate, 2xsaline sodium citrate (SSC) and placed on a slide for denaturation at 80 °C for 2 min, and hybridization at 54 °C for 30 min. After hybridization, cells were washed with 0.1 SSC at 65 °C for 3 × 10 min. Streptavidin Alexa Fluor 488 conjugate (Invitrogen) was then added and incubated for 4 h at 37 °C. Nuclei were counterstained with DAPI. Images were captured using a confocal laser-scanning microscope (Carl Zeiss).

### Tumor specimens and in situ hybridization

Human breast cancer tissue microarrays were purchased from the National Human Genetic Resources Sharing Service Platform2005DKA21300 (Shanghai Outdo Biotechnology Company Ltd., China). Tissue sections were deparaffinized and gradually rehydrated. After treatment withpepsin, tissues were hybridized with 5′biotin-labeled LNA^TM^-modified PAS1 probe (Exiqon, Vedbaek, Denmark) at 54 °C overnight. Subsequently, streptavidin was conjugated to Poly Horseradish Peroxidase (Poly-HRP) using the detection kit (SP-9002, Zhong Shan Jin Qiao, Beijing, China). Criteria for staining were classified into four groups based on staining level: no staining,0; low staining, 1+; moderate staining, 2+; and strong staining, 3+.

### Western blot and co-immunoprecipitation assays

Cells were harvested and washed once with cold PBS. Cells were then lysed in lysis buffer (50 mM Tris-HCl, 250 mM NaCl, 5 mM EDTA, 50 mM NaF, 0.1% NP40, and 1% protease inhibitor cocktail). For each immunoprecipitation assay, proteins were incubated with anti-HA antibody at 4 °C overnight, followed by incubation with protein A/G agarose beads (Santa Cruz Biotechnology). Beads were washed three times with NP40 buffer, the bound proteins were eluted with 2×SDS loading buffer and then boiled at 100 °C for 5 min. Precipitated proteins were detected using western blot. Antibodies against anti-DNMT1 (Abcam), anti-GAPDH (ZSGB-BIO), anti-SUV39H1 (Active Motif), anti-vigilin (Abcam), and anti-PH20 (Abcam) were used.

### Cell migration assay

A cell suspension containing 5 × 10^4^ cells was added to the upper chamber of a modified Boyden Chamber (BD Biosciences, San Jose, CA, USA), and 0.6 ml media containing 20% FBS was added to the lower chamber. After 12–24 h, non-migratory cells were removed by wiping the top of the membrane with cotton swabs, and cells that had passed through the membrane were fixed, stained with crystal violet, and counted by microscopy.

### Animal models and reagents

MDA-MB-231-Luc-D3H2LN cells were inoculated orthotopically onto the abdominal mammary fat pad or injected into tail vein of 6-week-old female NOD-SCID mice. Control RNA or the PAS1-30-nt RNA were chemically synthesized with modifications of 2′-O-methylation and 5′-Cholesterol from Ribobio Co. (Guangzhou, China) for in vivo RNA delivery. Control RNA or the PAS1-30-nt RNA (Ribobio) was injected into the tail vein of NOD/SCID mice twice a week for total 4 times. Decitabine (Sigma) was injected intraperitoneally at 2 mg/kg twice a week for total 4 times. The mice were then injected with D-luciferin abdominally (200 mg/kg, 10 min before imaging), and anesthetized; finally, images were taken using a Xenogen IVIS Lumina system (Caliper Life Sciences, Hopkinton, MA). Images were analyzed using the Living Image software ver. 3.0. (Caliper Life Sciences, Hopkinton, MA, USA). Photon flux was normalized to background.

### Ethics

The Ethics Committee of Peking University Health Science Center approved the mouse experiments (Permit Number: LA2014122) for this study. The mice were handled in accordance with the ethical standards of the Helsinki Declaration of 1975 and the revised version in 1983. We also referred to the procedures by Workman et al.^38^.

### Statistical analysis

All statistical analyses were performed using GraphPad Prism software. Two-tailed unpaired t-tests were used to analyze the two groups. One-way analysis of variance was used to analyze multiple groups. All results are presented as mean ± SEM. Statistical significance was set at *p* < 0.05.

## Supplementary information

Supplementary figures and legends

Table S1. Mass Spectrometry

Table S2. PAS1 co-expression

## Data Availability

All data supporting this paper are present within the paper and the Supplementary Materials. The original datasets are also available from the corresponding author upon request.
