# Supplementary material for: The DNMT1-PAS1-PH20 axis drives breast cancer growth and metastasis
Source: Signal Transduct Target Ther. 2022 Mar 21;7:81. doi: 10.1038/s41392-022-00896-1 (PMC8934873; doi:10.1038/s41392-022-00896-1)
Supplement: Supplementary file 1 — Supplementary figures and legends [file 41392_2022_896_MOESM1_ESM.doc]

**Supplementary Materials for**

**“The DNMT1-PAS1-PH20 axis drives breast cancer growth and metastasis”**

Yenan Fu^1#^, Xi Zhang^1#^, Xiao Liu^2#^, Peng Wang^1^, Wenhui Chu^1^, Wei zhao^1^, Yunling Wang^1^, Guangbiao Zhou^3^, Yu Yu^1^* and Hongquan Zhang^1^*

^1^ Department of Human Anatomy, Histology and Embryology, Key Laboratory of Carcinogenesis and Translational Research (Ministry of Education), State Key Laboratory of Natural and Biomimetic Drugs, Peking University Health Science Center, Beijing, 100191, China, ^2^Department of Orthopedics, Peking University Third Hospital, Beijing, 100191, China, ^3^State Key Laboratory of Molecular Oncology, National Cancer Center, National Clinical Research Center for Cancer, Cancer Hospital, Chinese Academy of Medical Sciences and Peking Union Medical College, Beijing 100021, China.

^#^ These authors contributed equally to this work

**Correspondence to:** Hongquan Zhang ([Hongquan.Zhang@bjmu.edu.cn](mailto:Hongquan.Zhang@bjmu.edu.cn)) and Yu Yu ([yuyu@bjmu.edu.cn](mailto:yuyu@bjmu.edu.cn)), Peking University Health Science Center, #38 Xue Yuan Rd., Beijing 100191, China. Tel: 86-1082802424; Fax: 86-1082802424.

**Conflicts of interests:** The authors declare no competing interests.

**This file includes:**

Supplementary Figures S1 to S8


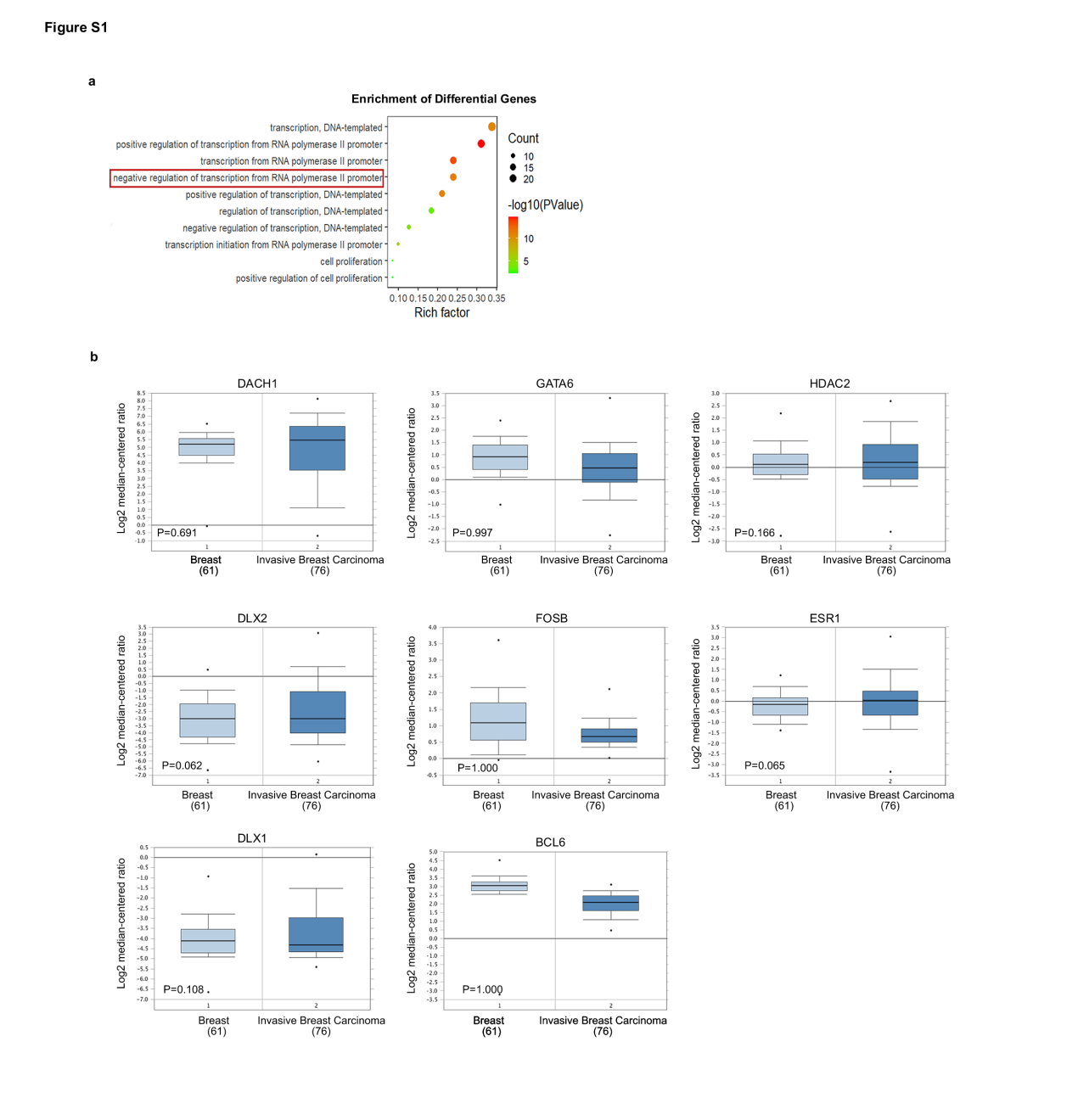


Figure S1. (a) Molecular function analysis of TFs with PAS1 fold change ≥ 2 or ≤ 0.5 using DAVID website (https://david.ncifcrf.gov/summary.jsp). (b) Analysis of expression of eight TFs in breast cancer patients' samples obtained from Oncomine datasets.


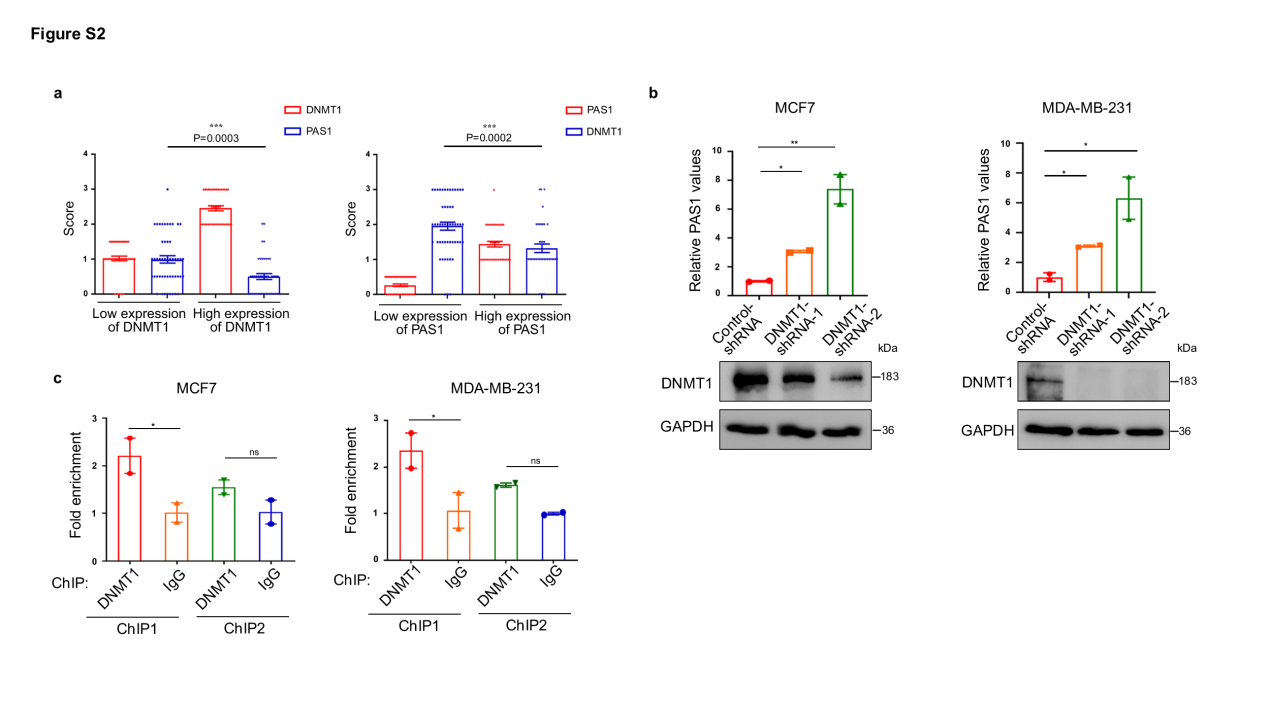


Figure S2. (a) Both PAS1 expression (CISH) and DNMT1 expression (IHC) were examined in paraffin-embedded tissues of 91 breast cancer patients. Analyses of expression level of PAS1 (DNMT1) in the two groups of high and low expression of DNMT1(PAS1). (b) Control and DNMT1 shRNAs were transfected, followed qRT-PCR and Western blot analysis. (c) Lysates from MCF7 and MDA-MB-231 cells were extracted for ChIP assays using anti-DNMT1 antibodies. Q-PCR assays were performed to quantify ChIP-enriched DNAs.


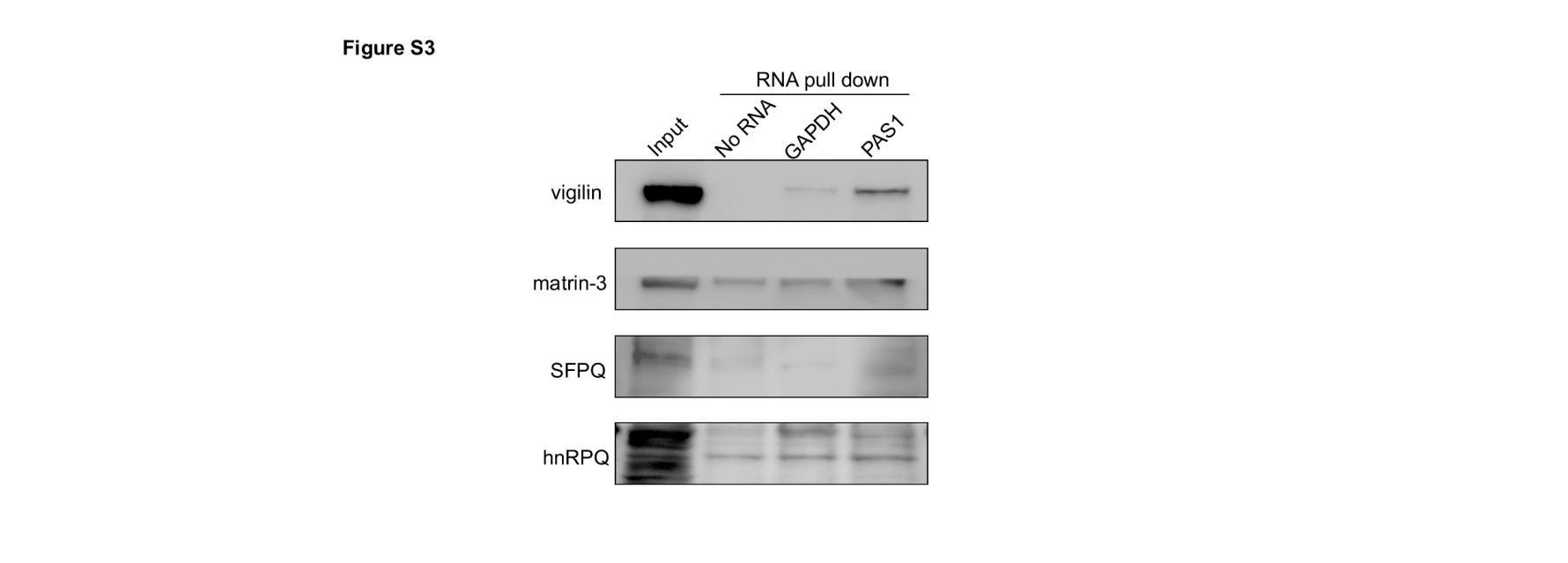


Figure S3. RNA pull down was performed to detect the interaction of PAS1 RNA and vigilin, matrin-3, SFPQ, and hnRPQ in MDA-MB-231 cells.


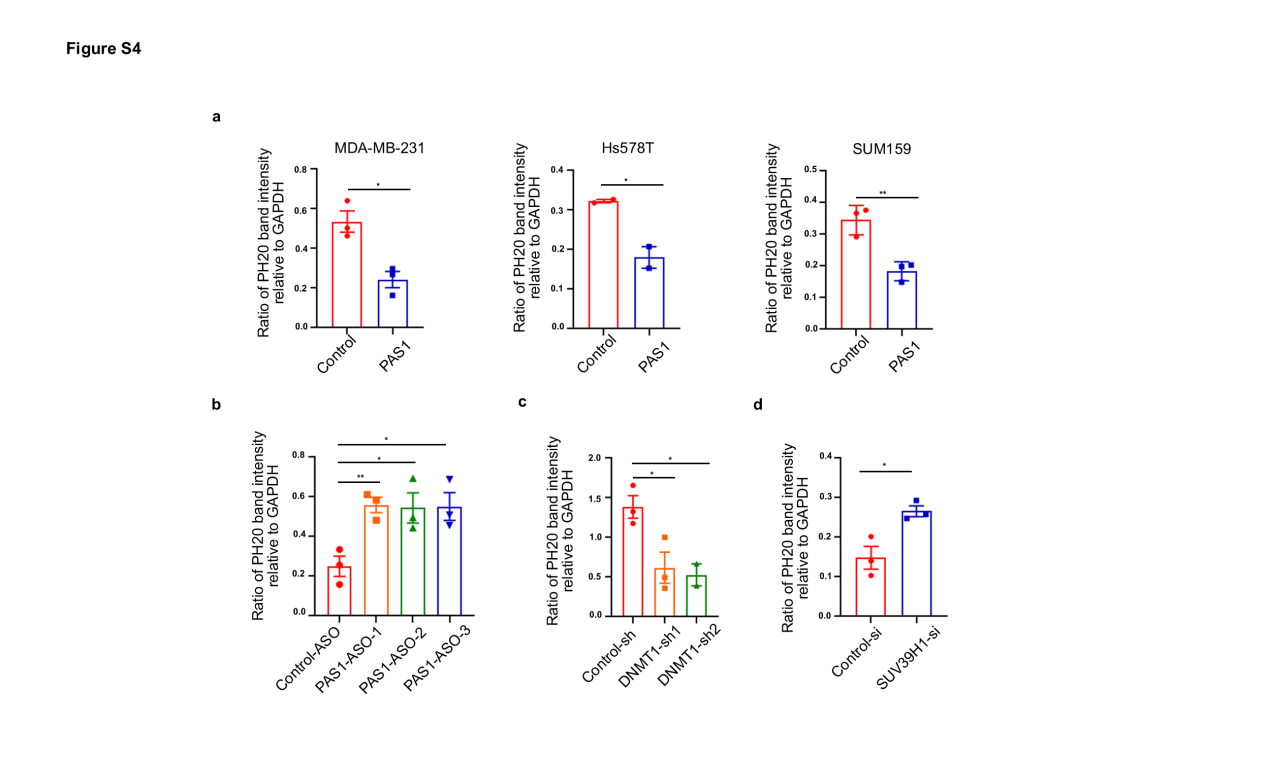


Figure S4. (a) An empty or PAS1-overexpressing vector was transfected into different breast cancer cells, followed by Western blot. (b) Control or PAS1 ASOs were transfected into MCF7 cells, followed by Western blot. (c) Lysates were prepared from MDA-MB-231-control/ DNMT1-shRNAs cells for Western blot. (d) SUV39H1 siRNA was transfected into MDA-MB-231 cells, followed by Western blot. All the PH20 protein bands in a, b, c, and d were scanned and relative band intensities were normalized to each GAPDH band. The column diagrams represent average relative band intensity with SEM from three independent experiments. Unpaired two-tailed *t* test was performed. *p < 0.05, **p < 0.01.


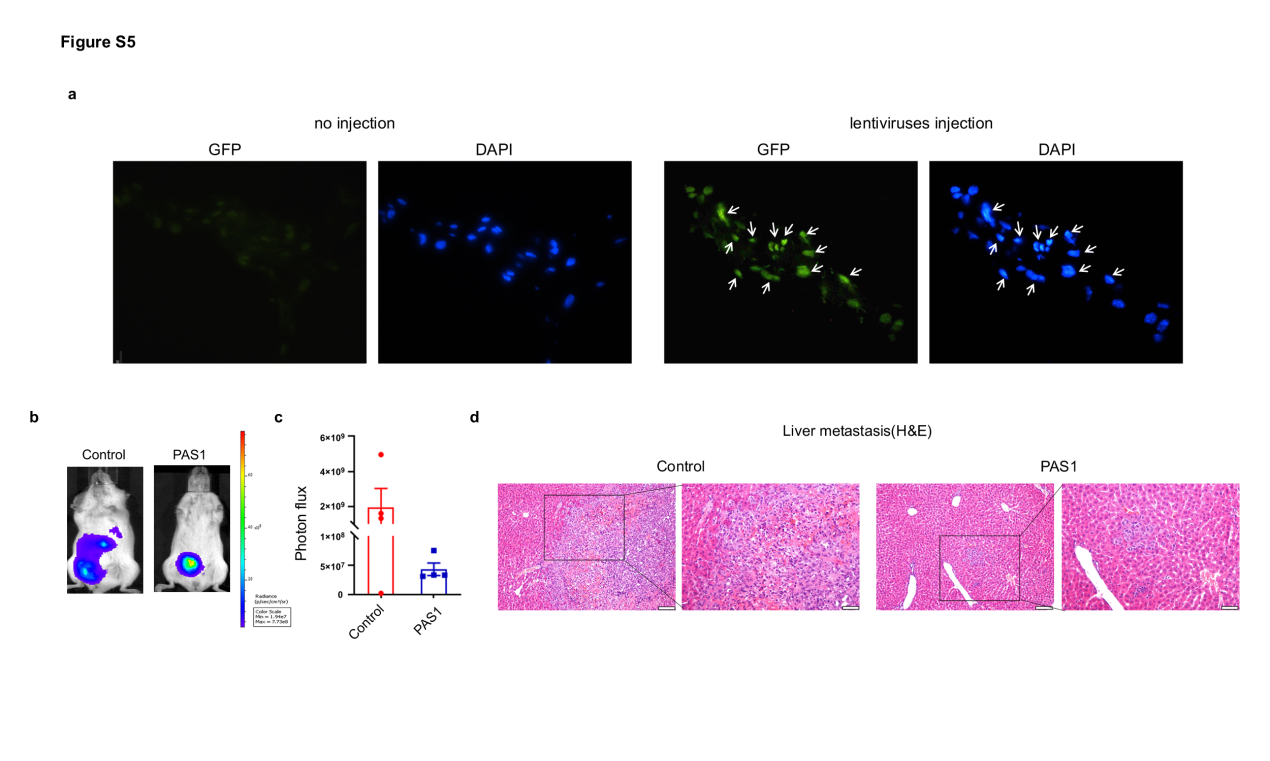


Figure S5. (a) Lentivirus (carrying EGFP gene) were injected into the mammary glands of 10 week-PyMT mice. After 48 hours, the mammary tissue was made into freezing sections to observe the GFP fluorescence. The GFP-positive cells were shown by the white arrows. (b) MDA-MB-231-Luc-D3H2LN cells with or without PAS1 were inoculated onto the abdominal mammary fat pad of mice. Representative bioluminescent images of primary tumor and liver metastasis. (c) Bioluminescence-based quantitation of liver metastasis (n=4/group; control group (3/4) and PAS1 group (0/4)). Data are means ±SEM by F test to compare variances. (d) Representative liver-metastasis specimens were sectioned and stained with H&E.


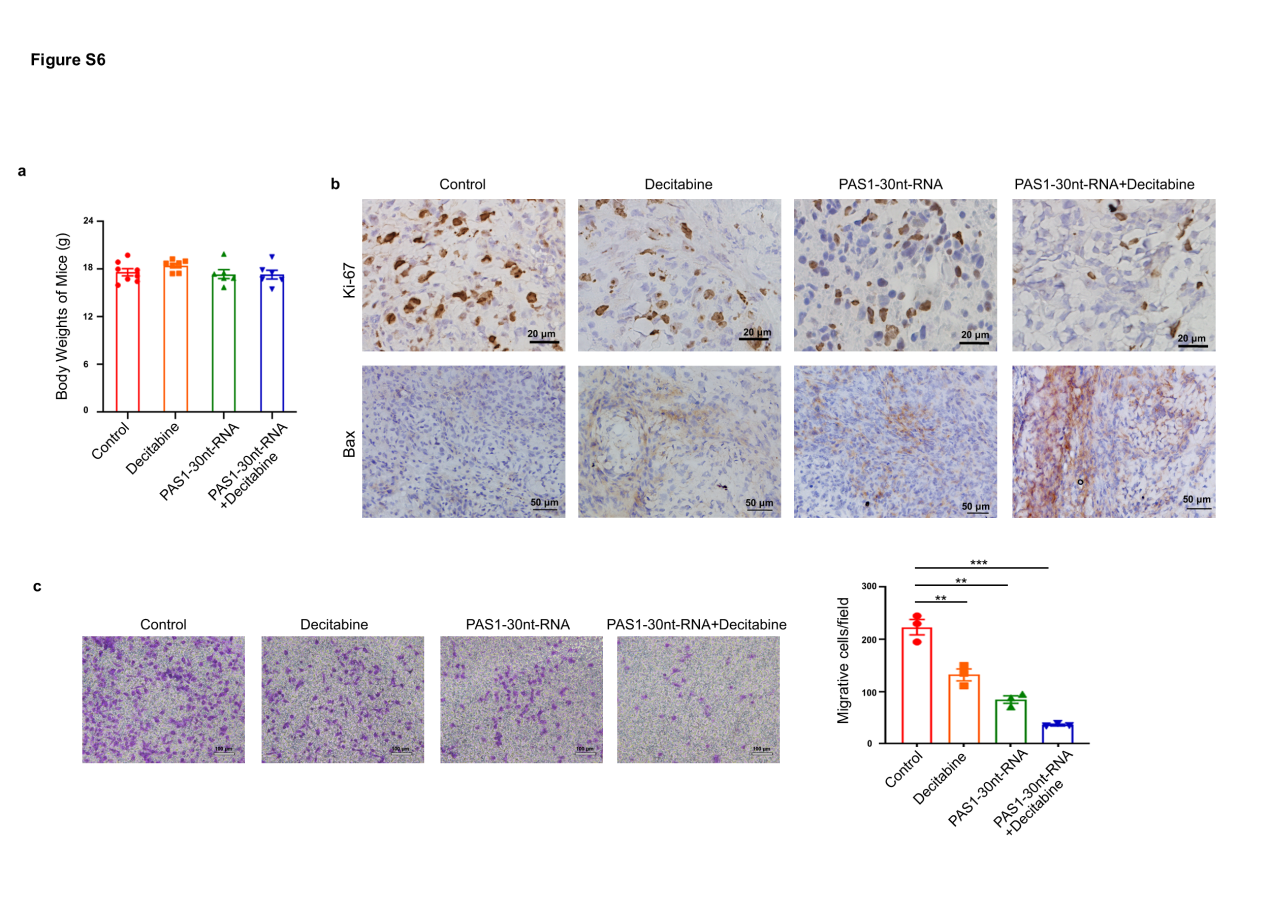


Figure S6. (a) Mice in the four groups were weighed up. (b) The tumor tissues of 4 groups were made into freezing sections, and stained by Ki67 and Bax for immuohistochemistry analysis. (c) Plasmids of control or PAS1-30nt overexpressing were transfected into MDA-MB-231 cells, with or without decitabine treatment (5 μM, 48 h). The 4-group cells were performed migration assay. Data are means ± SEM from three experiments by two-tailed *t* test.


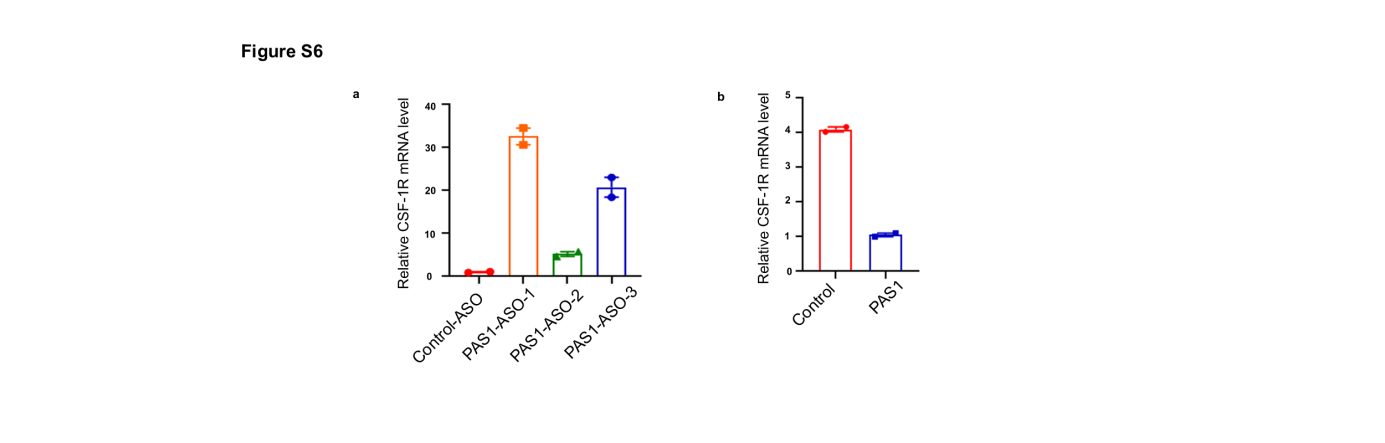


Figure S7. (a) Control or PAS1 ASOs were transfected into MCF7 cells, followed by qRT-PCR to detect the mRNA level of CSF-1R. (b) An empty or PAS1-overexpressing vector was transfected into MDA-MB-231 cells, followed by qRT-PCR to detect the mRNA level of CSF-1R.


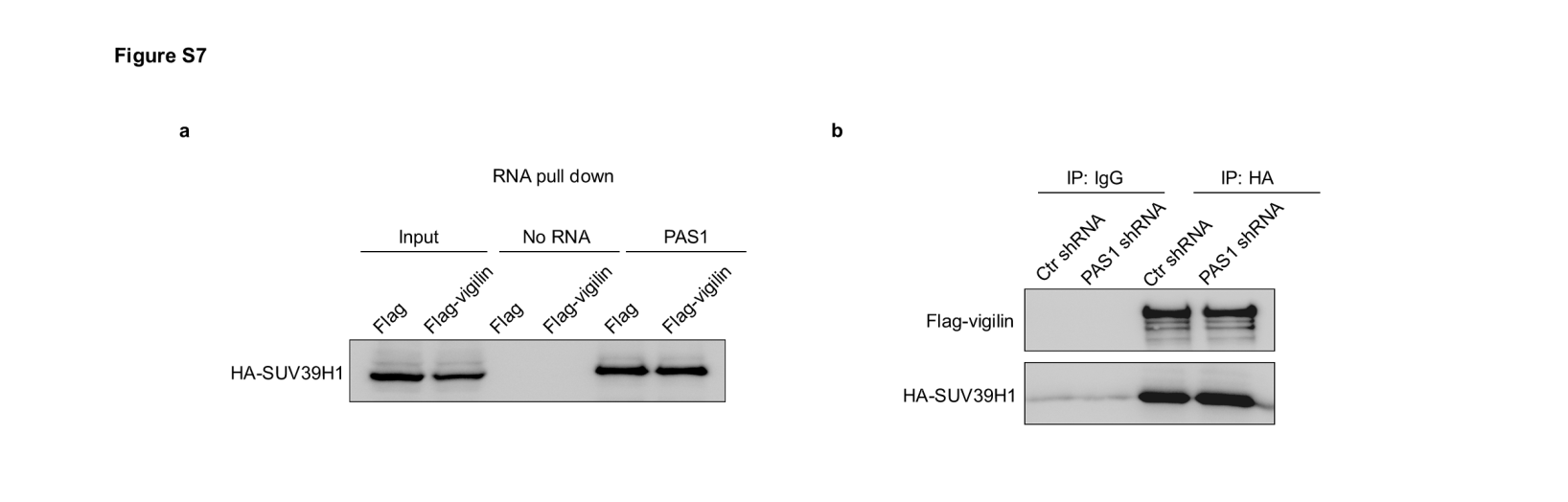


Figure S8. (a) Plasmids of expressing Flag or Flag-vigilin and HA-SUV39H1 were transfected into 293T cells. Biotin-labeled PAS1 was incubated with lysates from 293T cells, followed by RNA pull down analysis. (b) Plasmids of expressing Flag-vigilin, HA-SUV39H1, control or PAS1 shRNA were transfected into 293T cells. Lysates were extracted for Co-IP assay using the anti-HA antibody.
